# Gait biomechanics and postural adaptations in forward head posture: a comparative cross-sectional study

**DOI:** 10.1186/s12891-025-08882-8

**Published:** 2025-08-07

**Authors:** Guohao Lin, Xiong Zhao, Zhihao Tao, Weijie Wang

**Affiliations:** 1 The Third School of Clinical Medicine (School of Rehabilitation Medicine), Zhejiang Chinese Medical University, Hangzhou, China; 2 School of Science and Engineering, University of Dundee, Dundee, UK; 3 School of Human Kinetics, University of Ottawa, Ottawa, Canada; 4 Department of Orthopaedic and Trauma Surgery, School of Medicine, University of Dundee, Dundee, UK

**Keywords:** Forward head posture, Gait, Craniovertebral angle, Trunk flexion

## Abstract

**Background:**

Forward head posture (FHP) is a common postural deviation in the sagittal plane. Despite the growing interest in FHP, research on gait biomechanics in individuals with FHP remains scarce. This study aimed to investigate gait biomechanics in FHP, with a gait performance-based craniovertebral angle (CVA) cut-off.

**Methods:**

Forty-eight participants were included in the study, with CVA measurements used to assess head-and-neck posture. Three-dimensional kinematic and kinetic data were collected using a motion capture system during three walking trials at preferred speeds. Spatiotemporal gait parameters, joint angles, joint moments, joint powers, joint forces, center of mass (COM) trajectories, and COM-to-joint (knee and ankle) angles were analyzed. Time-series data were compared between the two groups using statistical parametric mapping to identify potential changes during the gait cycle.

**Results:**

Forty-eight participants were divided into control (*n* = 26) and FHP (*n* = 22) groups based on a CVA cut-off of 44 degrees determined by K-means clustering. There were no significant differences in spatiotemporal gait parameters between the control and FHP groups. However, the FHP group exhibited significantly increased trunk flexion during the loading response and initial midstance (2.21–14.50%, *p* = 0.047), as well as pre-swing and initial swing phases of the gait cycle (46.45–68.86%, *p* = 0.039). The COM-to-knee angle was significantly reduced during mid-swing in the FHP group (71.26–87.92%, *p* = 0.007). Additionally, significant differences in sagittal knee joint power and longitudinal joint forces at the knee and ankle were observed in the final stages of the gait cycle (*p* < 0.05). No significant differences were found in COM trajectories or other gait parameters.

**Conclusion:**

This study identified phase-specific compensatory trunk flexion in individuals with FHP, despite preserved overall gait characteristics. A CVA cut-off of 44 degrees was proposed as a criterion for diagnosing FHP based on walking performance. These findings suggest that individuals with FHP employ specific biomechanical adaptations to maintain gait stability and underscore the importance of considering biomechanical adaptations in FHP diagnosis.

**Supplementary Information:**

The online version contains supplementary material available at 10.1186/s12891-025-08882-8.

## Background

Modern lifestyles and work environments, increasingly dominated by prolonged screen use and sedentary behaviors, have contributed to a rise in postural deviations, particularly forward head posture (FHP) [1, 2]. FHP, characterized by an anterior displacement of the head relative to the shoulders, is a common postural deviation in the sagittal plane [3, 4]. It is commonly quantified using the craniovertebral angle (CVA), with smaller CVA values indicating greater severity [5]. Previous studies have highlighted a significant relationship between altered sagittal spinal alignment, impaired postural control, and increased fall risk [6–8]. Specifically, individuals with FHP have demonstrated reduced vestibular and proprioceptive function, which are critical sensory inputs required for maintaining postural stability [9, 10].

Normal walking gait depends on effective postural control, environmental adaptability, and adequate body forward propulsion [11]. Therefore, deficits in sensory function and postural control associated with FHP could potentially disrupt normal walking patterns, leading to compensatory strategies to maintain gait stability [12]. These adaptations may contribute to inefficient gait patterns, subsequently reducing physical activity and elevating fall risk, with broader implications for health and mortality [13].

Despite these potential clinical implications, limited research has explored the gait biomechanics associated with FHP. A recent systematic review underscored this research gap, reporting scarce studies examining gait alterations in individuals with FHP and emphasizing the lack of standardized diagnostic criteria [12]. Current diagnostic methods for FHP often rely on subjective assessments, such as postural characteristics associated with specific conditions (e.g., headaches) [14], or visual assessments of ear-to-shoulder alignment [5]. Relying solely on subjective symptoms or visual assessment seems arbitrary without accounting for potential performance deficits associated with FHP.

Therefore, this study aimed to determine whether individuals with FHP exhibit gait biomechanical adaptations during normal walking and to propose a gait performance-based CVA cut-off as an objective diagnostic criterion. We hypothesize that individuals with FHP will adopt specific adaptations during walking to maintain stability and efficiency despite the altered head posture. The findings of this study are intended to enhance the understanding of biomechanical adaptations associated with FHP and support the development of objective clinical assessment methods.

## Methods

This study was conducted at the Institute of Motion Analysis and Research, University of Dundee, UK. Ethical approval was obtained from the Schools of Medicine and Life Sciences Research Ethics Committee, University of Dundee (SMED REC No. 22/38).

### Recruitment and participants

Participants were recruited through verbal invitations and poster advertisements, with eligibility criteria including healthy adults aged 18–70 years with no current injuries or neurological disorders. Exclusion criteria included musculoskeletal conditions of the spine (e.g., spinal deformities, scoliosis), and any conditions impairing balance and gait (e.g., neurological disorders, vestibular disorders, visual impairment, severe osteoarthritis, or acute pain during walking). Informed consent was obtained from all participants prior to participation.

### Anthropometric measurements and participant preparation

Anthropometric data were collected for model building and outcome normalization, following the VICON Nexus User Guide. Measurements included body mass, height, shoulder offset, elbow width, wrist width, hand thickness, distance between anterior superior iliac spines, leg length, knee width, and ankle width.

**Fig. 1:**
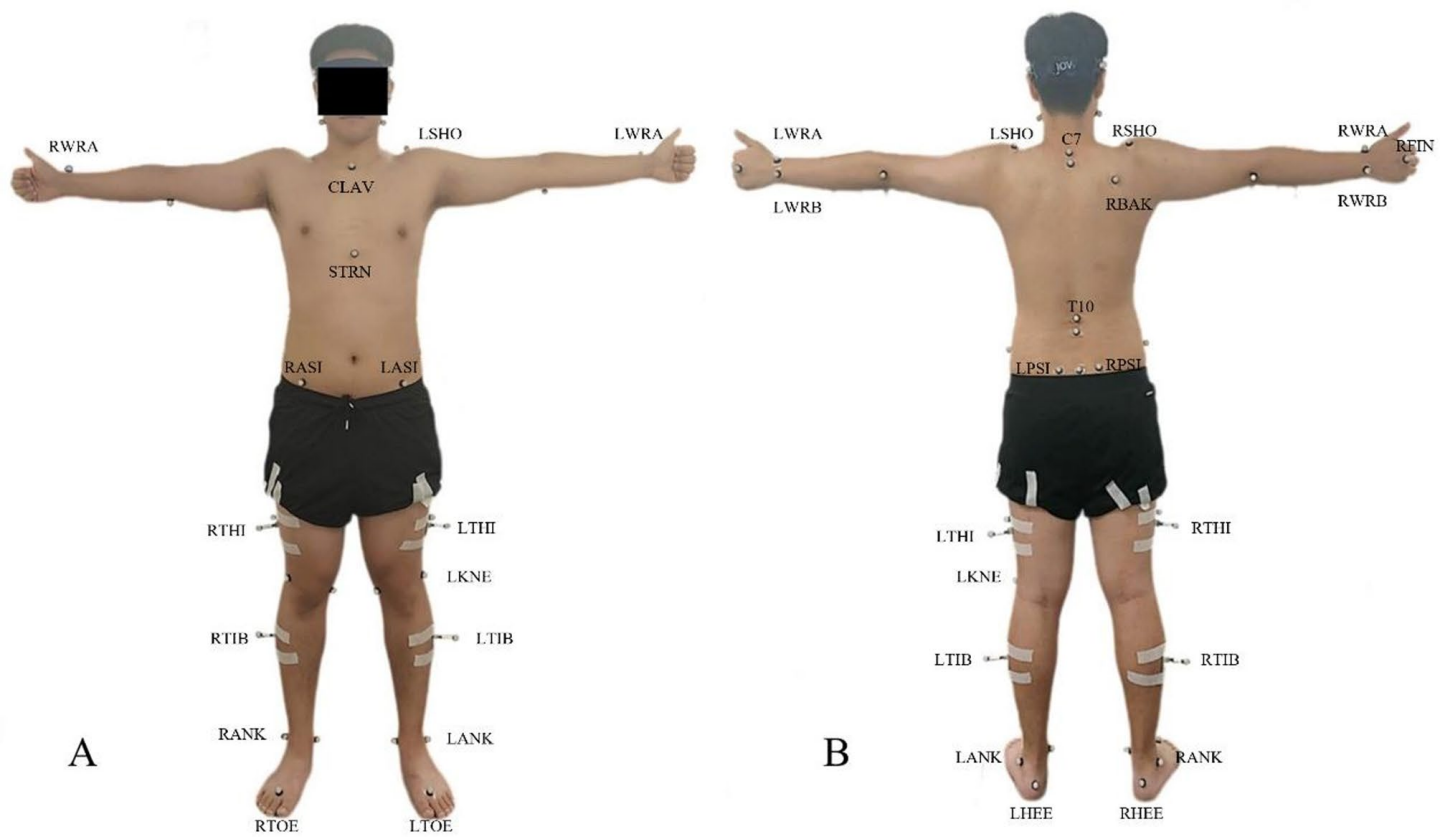
Marker placement (52 retro-reflective markers) on a participant in a T-pose. (**A**) The anterior view; (**B**) The posterior view

Upon arrival, participants completed a 3-minute familiarization session by walking naturally to determine their preferred walking speed. Male participants wore sports shorts, and females wore sports shorts and bras. All assessments were conducted barefoot. A total of 52 retro-reflective markers (14 mm in diameter) were placed at anatomical landmarks, including the head, trunk, pelvis, and upper and lower limbs (Fig. 1). Details of the full-body marker set, including marker names, definitions, and positions, are provided in the Supplementary Table. A static calibration trial was performed in a T-pose.

### Data collection and processing

Three-dimensional kinematic and kinetic data were captured using a 15-camera motion capture system (Vicon, Oxford Metrics Ltd., Oxford, UK) at 200 Hz, along with four ground-mounted force plates (60 × 40 cm, Advanced Mechanical Technology Inc., Watertown, MA, USA) at 1000 Hz. Participants walked continuously at their preferred speed until three successful gait trials were recorded, with each trial defined by two consecutive right heel strikes detected using a vertical ground reaction force threshold of 10 N. The use of three trials with a continuous walking protocol was chosen to enhance reliability, account for natural variability in gait, and allow participants to establish a steady walking speed [15], as determined during a 3-minute familiarization session.

Motion capture data were processed in Vicon Nexus (Version 2.12), and marker trajectories and force plate data were exported to Visual3D software (C-Motion, Inc., Germantown, MD, USA). A full-body model was created based on the static calibration trial using standardized Visual3D procedures. Spatiotemporal parameters, joint angles, moments, powers, and forces for the hips, knees, and ankles were calculated, as well as trunk joint angles and the whole-body center of mass (COM). Flexion/extension was about the x-axis, adduction/abduction was about the y-axis, and rotation was about the z-axis.

Kinematic and kinetic data were filtered using a fourth-order low-pass Butterworth filter with a 10 Hz cut-off. All data were time-normalized to 101 points representing the gait cycle (0–100%). Walking speed and stride length were normalized by individual right leg length, the vertical COM trajectory by height, and kinetic outcomes by body mass.

### K-means clustering analysis

To address the absence of standardized diagnostic criteria for FHP, K-means clustering analysis was used to classify participants into two groups, control group and FHP group. This machine-learning approach is widely utilized in postural control and gait studies to identify homogeneity within a population and to establish objective cutoff points [16, 17]. In our analysis, we implemented the K-means algorithm in MATLAB with the number of clusters (k) set to two, corresponding to a binary classification of FHP presence or absence. The algorithm minimized the sum of squared Euclidean distances between data points and the centroids of their respective clusters. The clustering process involved iterative reassignments of points to the closest centroid until convergence, ensuring clear separation between groups.

Three input variables were used in the clustering process: mean CVA, normalized walking speed, and normalized stride length. These parameters were chosen for their biomechanical and clinical significance. Mean CVA served as the primary indicator of FHP severity, while normalized walking speed and stride length provided time- and anthropometry-adjusted measures of gait performance. Normalized walking speed is a key gait parameter with time-normalization properties that enhance inter-participant comparability. Normalized stride length is associated with the moment arm of gravity acting on the knee and ankle joints, reflecting biomechanical adaptations during walking. The cutoff value for diagnosing FHP was determined by the boundary between these two clusters, based on their respective CVA values. Participants whose CVA values fell below this boundary were classified as having FHP.

### Forward head posture assessment and COM-to-joint angle

The CVA was measured in the sagittal plane using a motion capture system. It was defined as the angle formed between the line connecting the C7 spinous process to the midpoint of the two tragus markers and the horizontal plane. A smaller CVA indicates a greater severity of FHP. Furthermore, one frame only captures a moment of the dynamic posture during walking, whereas a mean CVA value over a period is more representative for posture assessment. Thus, the mean CVA of each participant was calculated during the three successful gait trials.

**Fig. 2:**
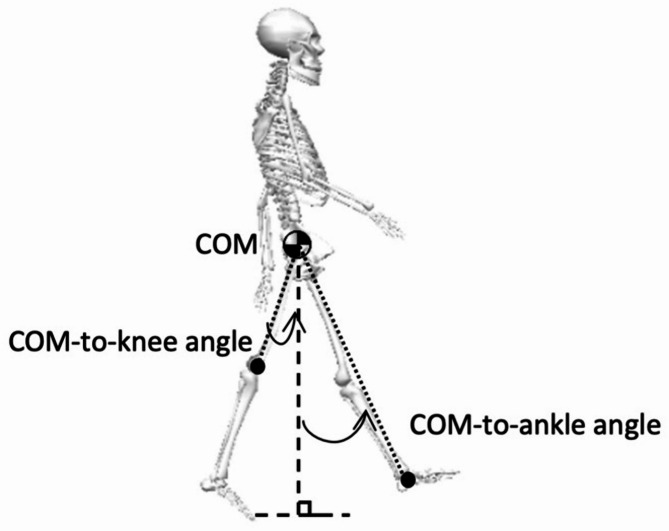
The COM-to-knee and the COM-to-ankle angles. The positive direction is assigned to the counter-clockwise rotation. COM: center of mass

To maintain the optimal COM trajectory during gait, knee and ankle kinematic mechanisms play an important role in smoothing the displacement of the COM in the sagittal plane, which improves gait efficiency and energy exchange. The COM-to-knee and COM-to-ankle angles in the sagittal plane reflect the perpendicular distance between the gravity line of the COM and the joint rotation axes (i.e., moment arm) [18]. Therefore, COM-to-knee and COM-to-ankle angles during walking were additionally analyzed in this study. The COM-to-knee (or -ankle) angle is defined as the sagittal plane orientation of the vector from COM to the knee (or ankle) rotations axis, with the vertical line of COM as the reference (Fig. 2). The knee and ankle rotation axes in this study were determined using the knee joint marker and ankle joint marker, respectively.

### Statistical analysis

Statistical analysis was conducted using SPSS (version 26, IBM, USA). The Shapiro-Wilk test was used to assess normality. For comparisons between the FHP and control groups, independent t-tests were used for normally distributed data, while the Mann-Whitney U test was applied to non-normally distributed data. In terms of the time-series gait kinematic and kinetic data, the statistical parametric mapping (SPM) analysis method was used for statistical analysis, using the spm1d package. The SPM has been suggested as a suitable method for analyzing time-series data, using the random field theory to account for data variability [19]. Therefore, this study presented statistical results over the entire gait cycle instead of comparing a specific event, which is conducive to identifying potential changes in walking gait. A significance level of 0.05 was set for statistical analysis.

## Results

### Demographics and cut-off CVA for grouping

The 48 participants were divided into control (*n* = 26, 9 males/17 females) and FHP (*n* = 22, 10 males/12 females) groups based on a cut-off CVA value of approximately 44 degrees. Demographic characteristics and spatiotemporal gait parameters for both groups are summarized in Table 1. No statistically significant differences were observed between the two groups in demographic variables (*p* > 0.05), but the FHP group had a significantly smaller CVA (*p* < 0.001). Additionally, no statistically significant differences were found in spatiotemporal gait parameters between the groups.

**Table 1:** The demographics and Spatiotemporal gait parameters of the control and FHP groups

Characteristics	Control group (*N* = 26) Mean ± SD / Median ± IQR	FHP group (*N* = 22) Mean ± SD / Median ± IQR	*p*-value
CVA (degree)	47.20 ± 4.78	41.91 ± 4.06	< 0.001*^m^
Body mass (kg)	57.50 ± 13.90	64.30 ± 22.90	0.19^m^
Height (cm)	166.66 ± 8.74	167.62 ± 9.56	0.72
Leg length (cm)	85.83 ± 5.72	86.60 ± 7.04	0.68
Age (year)	26.00 ± 6.50	26.00 ± 7.25	0.89^m^
BMI	21.71 ± 3.15	23.10 ± 3.59	0.16
Sex	9 M / 17 F	10 M / 12 F	
Stride length (m)	1.25 ± 0.10	1.25 ± 0.09	0.97
Normalized stride length	1.45 ± 0.10	1.44 ± 0.10	0.75
Walking speed (m/s)	1.15 ± 0.12	1.18 ± 0.12	0.32
Normalized walking speed	1.35 ± 0.16	1.37 ± 0.27	0.63^m^
Stride duration (s)	1.09 ± 0.07	1.06 ± 0.08	0.15
Walking cadence (steps/minute)	108.98 ± 7.38	112.87 ± 8.83	0.10

*: *p* < 0.05; BMI: body mass index; M: male; F: female; SD: standard deviation; FHP: forward head posture; IQR: interquartile range; m: Mann-Whitney U test

### Trunk angle and lower limb joint angles

**Fig. 3:**
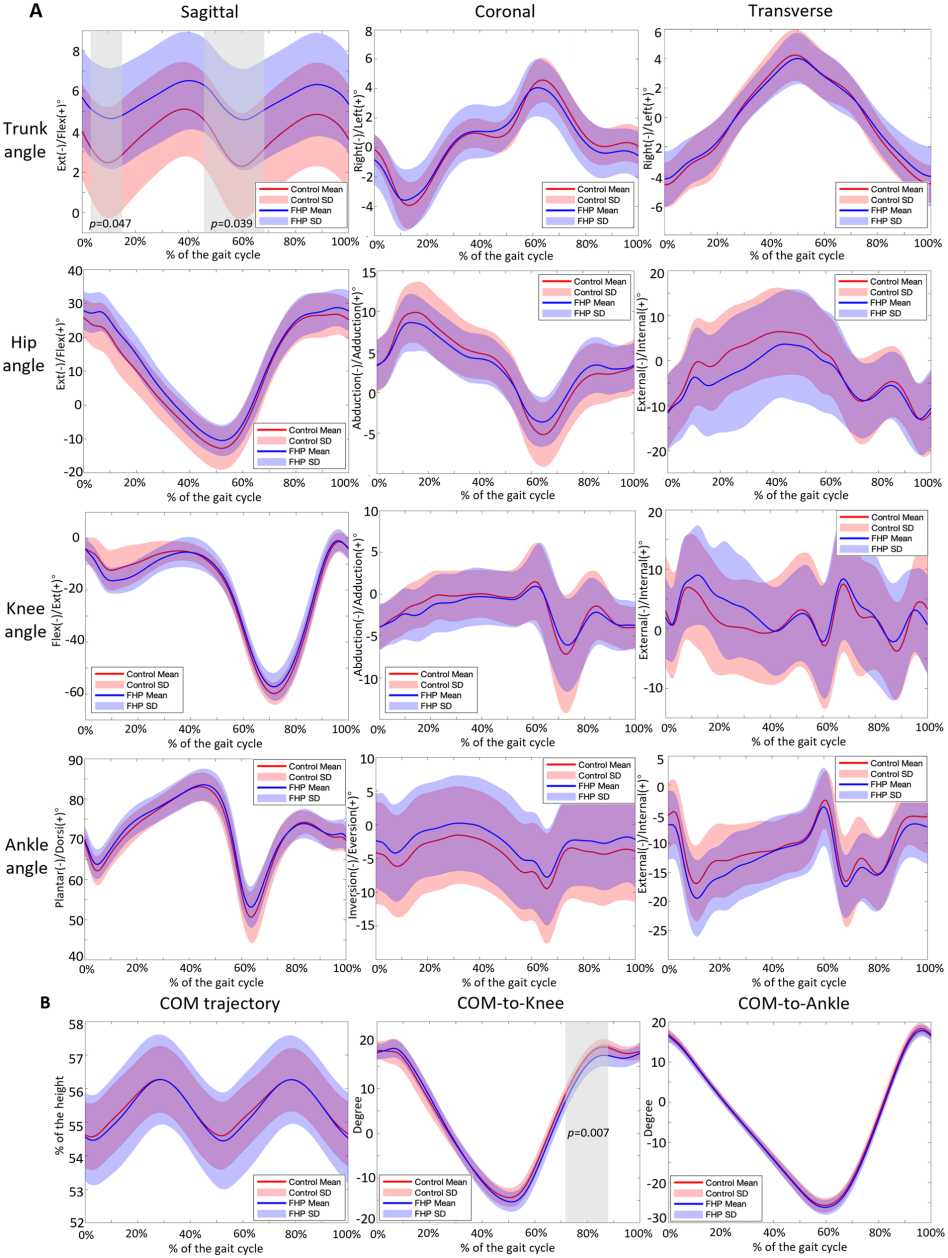
Gait kinematics comparison between the control and FHP groups. (**A**) Comparison of the trunk, hip, knee, and ankle joint angles between the control and FHP groups. (**B**) Comparison of the COM trajectory, COM-to-knee, and COM-to-ankle joint angle. Solid lines: mean value, red and purple shaded bands: standard deviation, grey shaded bands: regions with significant differences. The alpha level was set at 0.05

SPM analysis revealed significant differences in the sagittal trunk angles between the FHP group and the control group in two regions: 2.21–14.50% (*p* = 0.047) and 46.45–68.86% of the gait cycle (*p* = 0.039). No significant differences were observed in the coronal or transverse trunk angles (*p* > 0.05), indicating no changes in trunk lateral flexion or rotation. In the comparison of lower limb joint angles during the gait cycle, no statistically significant differences were observed between the FHP group and the control group (*p* > 0.05) (Fig. 3).

### COM trajectory and COM-to-joint angle

There were no significant differences in the vertical COM trajectory between the two groups (*p* > 0.05). However, the sagittal COM-to-knee angle showed a significant difference between 71.26% and 87.92% of the gait cycle (*p* = 0.007), while the sagittal COM-to-ankle angle did not show any significant difference (*p* > 0.05).

### Lower limb joint moment

The SPM analysis of lower limb joint moments did not reveal significant differences between the FHP and control groups (Supplementary Fig. 1).

### Lower limb joint power

**Fig. 4:**
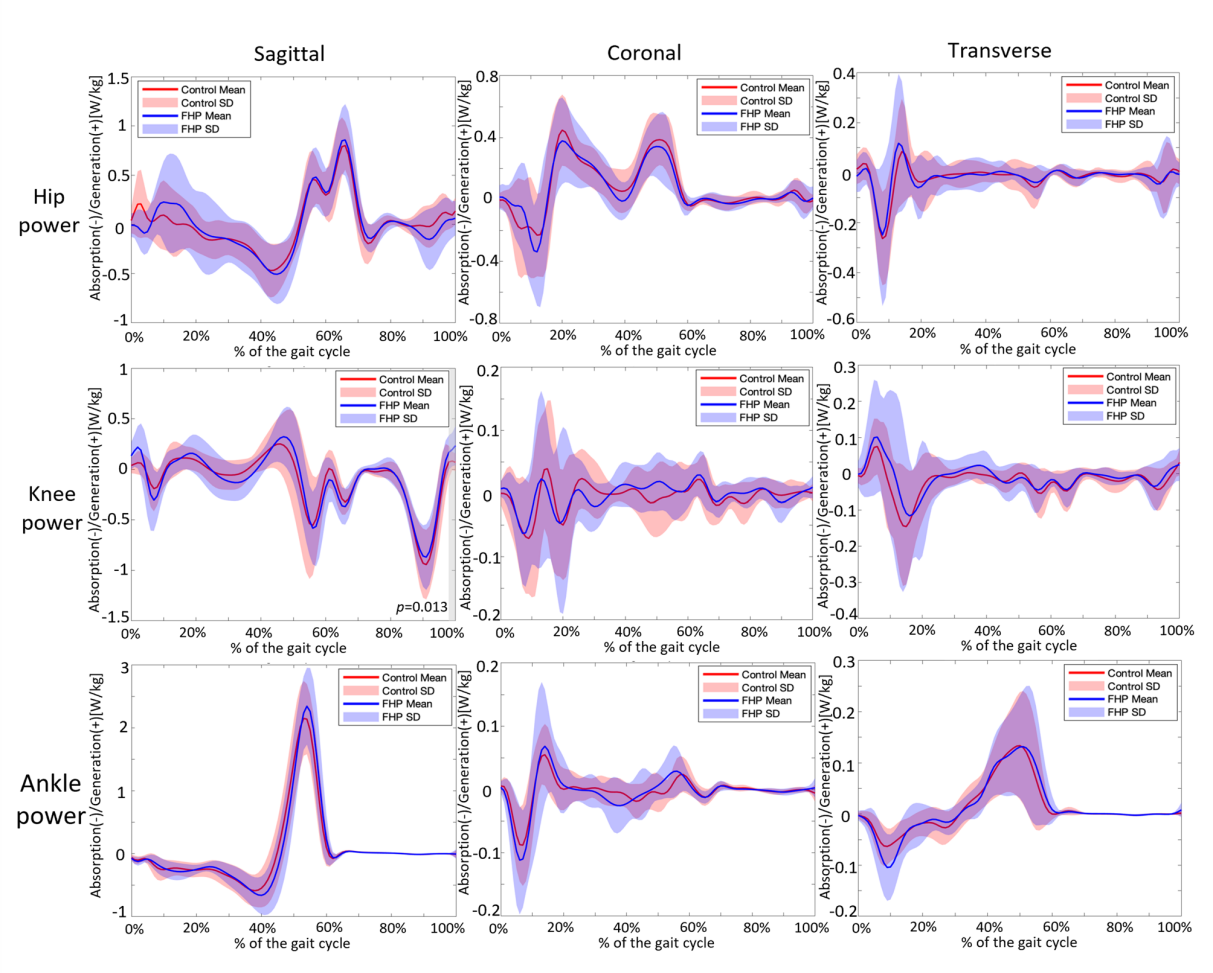
Comparison of the hip, knee, and ankle joint powers between the control and FHP groups. Solid lines: mean value, red and purple shaded bands: standard deviation, grey shaded bands: regions with significant differences. The alpha level was set at 0.05

Lower limb joint power did not significantly differ between groups (*p* > 0.05), except for the sagittal knee joint power, which showed a significant difference between 99.48% and 100% of the gait cycle (*p* = 0.013, Fig. 4).

### Lower limb joint force

**Fig. 5:**
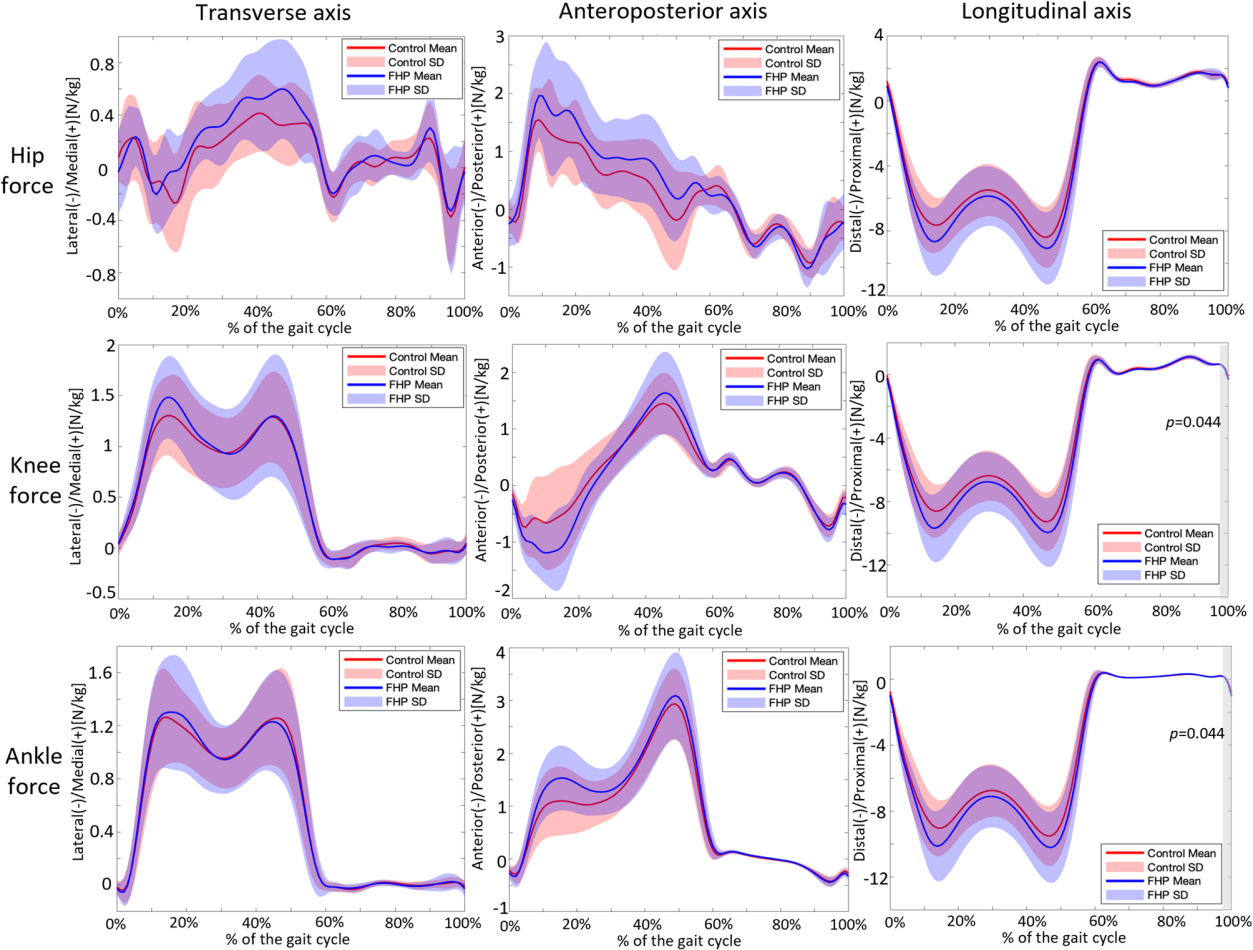
Comparison of the hip, knee, and ankle joint forces between the control and FHP groups. Solid lines: mean value, red and purple shaded bands: standard deviation, grey shaded bands: regions with significant differences. The alpha level was set at 0.05

Significant differences were found in knee and ankle joint forces along the longitudinal axis between 98.94% and 100% of the gait cycle (*p* = 0.044, Fig. 5). No other significant differences were identified for lower limb joint forces.

**Fig. 6:**
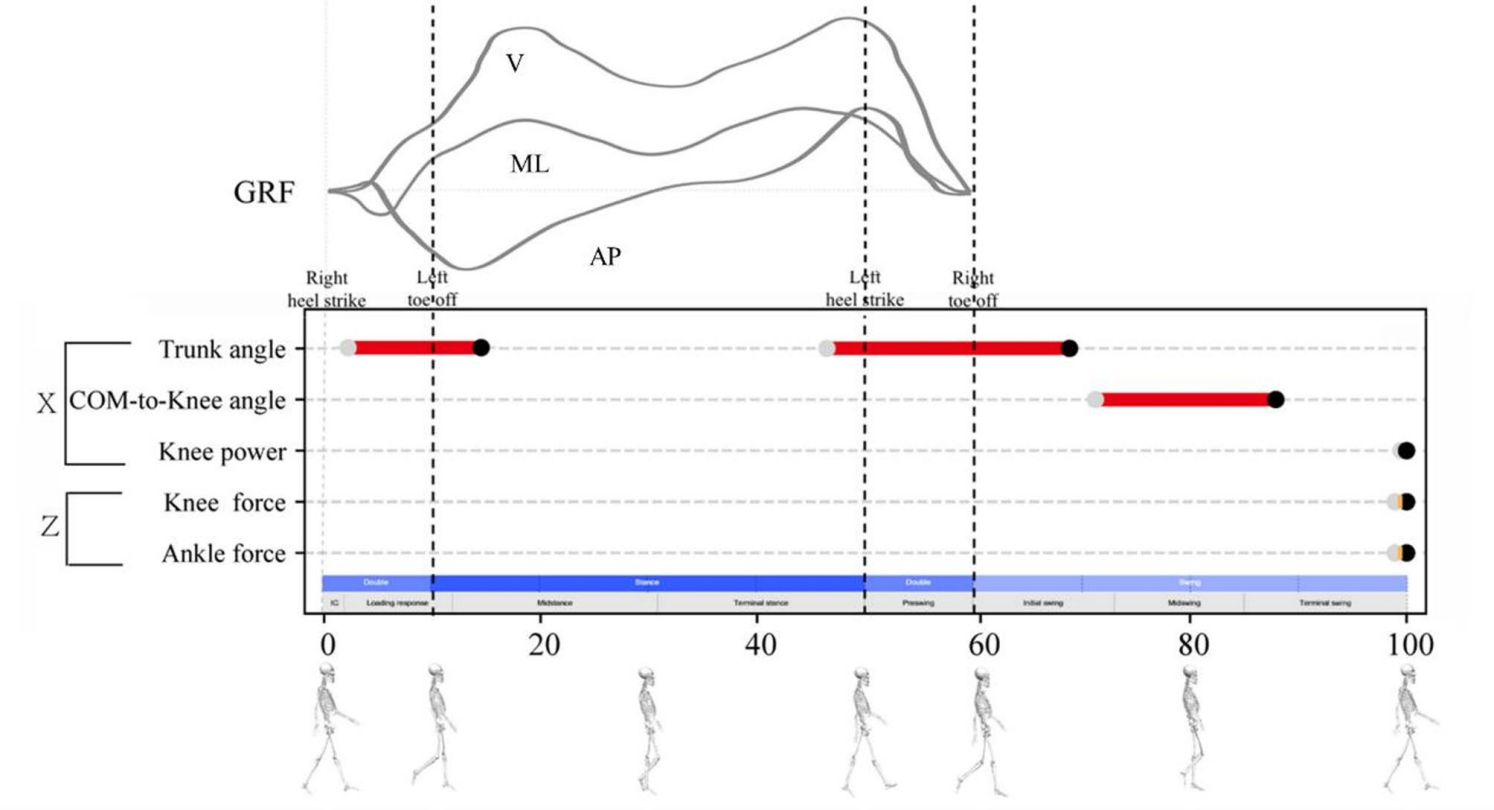
Regions with significant differences in the comparisons between the FHP and control groups during the gait cycle, starting from a right heel strike event. The red line indicates differences in the X plane (i.e., the sagittal plane), and the yellow line indicates differences in the Z plane (i.e., the transverse plane). Grey and black circles represent the onset and end of differences, respectively. GRF: ground reaction force. AP: anteroposterior; ML: mediolateral; V: vertical

The regions with significant differences in comparisons between the FHP and control groups are summarized in Fig. 6.

## Discussion

This study aimed to establish a gait performance-based criterion for FHP and investigate whether participants with FHP exhibit biomechanical adaptations during walking at a preferred speed. This study proposed a CVA value of 44 degrees and revealed significant differences in the trunk and lower limb joint parameters, while the FHP and control groups had similar spatiotemporal gait parameters and vertical COM trajectories, as well as comparable kinetic and kinematic characteristics in the coronal plane during the gait cycle.

Significant increases in trunk flexion were observed in the FHP group during the loading response, initial midstance, pre-swing, and initial swing phases of the gait cycle compared to the control group. This finding suggests that the participants with FHP adopted a more flexed trunk position during weight acceptance and transfer, as well as propulsion force generation, since the loading response and initial stage of the midstance phases are responsible for the weight acceptance and transfer, while the pre-swing phase is crucial for generating propulsion forces [20]. Furthermore, the observed increase in trunk flexion in the FHP group coincided with the two peaks of hip, knee, and ankle longitudinal joint forces. Previous research involving healthy participants who simulated increases in trunk flexion of 5 and 10 degrees during walking reported significant increases in hip and ankle joint moments, knee flexor muscle activity, and joint contact loads at the first peak of knee force [21]. These findings highlight the potential biomechanical implications of increased trunk flexion in individuals with FHP. However, the differences in joint moments observed in our study were not statistically significant. This discrepancy could be attributed to the relatively small difference in trunk flexion angles between the two groups, which was less than 5 degrees. Therefore, the modest forward lean of the trunk observed in FHP individuals may represent a deliberate or subconscious compensatory strategy aimed at counterbalancing the head’s forward displacement, thereby maintaining the COM over the base of support during walking.

Regarding the COM-to-knee angle, the FHP group exhibited significant differences during the mid-swing phase. This decrease, however, is less likely to affect the walking function, as the lower limb is swinging forward during this period. The lower limb did not need to adapt to the changes in the moment arm between the vertical COM gravity line and the knee joint axis. The absence of significant differences in joint angles and vertical COM trajectories suggests that the observed decrease in the COM-to-knee angle may be attributed to the anterior translation of the COM caused by FHP [22].

Additionally, three kinetic parameters of the FHP group differed from those of the control group during a similar period around the second heel strike event (Fig. 6). The knee joint of the FHP group exhibited significantly greater power during 99.48–100% of the gait cycle (Fig. 4), reflecting increased concentric knee extensor activity. We hypothesize that this increase is primarily driven by the quadriceps femoris, as muscle-driven dynamics simulations identified the vastus lateralis, vastus intermedius, and vastus medialis as the primary knee extensors activated during the terminal swing phase [23]. Additionally, a surface electromyography study revealed that the rectus femoris was also activated during this phase [24]. This increased activity plays a key role in stabilizing the knee joint for heel strike by enhancing knee stiffness [25].

The significantly greater knee and ankle forces along the longitudinal axis observed during the similar period (Fig. 6) indicate increased joint loading, which occurs slightly prior to the rise in knee power. This suggests that the increased concentric knee extensor activity may serve as an adaptive mechanism to manage the increased joint loading and ensure controlled limb positioning for a smooth transition into the stance phase. However, it is important to note that these modeled forces represent net forces, which include contributions from muscle forces, segmental inertia, and external forces, and are not equivalent to physiological joint reaction forces. Therefore, the interpretation of increased joint loading should be made with consideration of these methodological limitations.

Overall, trunk flexion appears to serve as a compensatory strategy to maintain normal spatiotemporal gait parameters and vertical COM trajectory in participants with FHP. This increased trunk flexion, particularly occurs during weight acceptance and propulsion force generation, corresponding to the anteroposterior GRF peaks during walking. The adaptation strategy of flexing the trunk was also observed during load carriage [18]. The study by Caron et al. [18]. demonstrated that the trunk forward lean was positively correlated with load carriage ranging from 0 to 40% body weight and played a substantial role in maintaining the amplitude of the vertical COM trajectory. They further inferred that the trunk forward lean may contribute to maintaining the optimal COM-to-knee and -ankle angles, thereby minimizing moment increases at the knee and ankle joints, particularly at push-off. The sufficiency of these trunk flexion adaptations to compensate for severe FHP remains uncertain, warranting further investigation. In particular, we observed several obvious variations in the kinematics and kinetics comparisons between the two groups, yet they did not reach a significant level.

This study proposed a CVA cut-off based on walking performance as an objective criterion for FHP diagnosis. According to our systematic review [12], CVA measured via photogrammetry in the lateral view is the most commonly used method to quantify FHP. However, there are various cut-off points of CVA in the previous studies, including 53 degrees [26, 27], 50 degrees [7], 49 degrees [28], and 48 degrees [29]. This disparity causes bias in comparisons between different studies. As mentioned above, previous FHP diagnostic criteria were based only on subjective pain or manual observation, rather than considering other potential performance deficits related to FHP. Notably, the mean CVA values of adults also vary across different assessment tasks [30]. Therefore, for studies aiming to determine whether individuals at risk of FHP have alterations in gait pattern, the gait performance-based criterion (CVA = 44 degrees during walking) should be adopted. Nevertheless, it is important to interpret the findings of this study in the context of CVA as a sagittal postural metric, rather than a comprehensive clinical definition of FHP. Further studies should consider integrating additional postural and neuromuscular assessments to characterize FHP subtypes.

There are several limitations in this study. First, the relatively narrow age range of participants (primarily 20–30 years old) limits the generalizability of the findings. In general, older adults may differ in their neuromuscular system attributes [31], and age is one of the important factors affecting gait [32]. Second, only the kinematics and kinetics of the right lower limb were analyzed in this study. Third, the absence of prior studies reporting on the gait kinematic and kinetic attributes of adults with FHP impedes direct comparison with existing literature. Fourth, this study did not quantify other factors that may influence gait, such as muscle strength, joint range of motion, and full-spine postural profiling, which should be considered in future research.

Accordingly, the recommendations for future research include the following aspects. First, future studies should explore gait biomechanics in real-world conditions to understand the functional implications of FHP in daily activities. Such studies could reduce potential biases from laboratory-based assessments and allow for more ecologically valid findings. Second, longitudinal studies are essential to trace the progression of FHP-related biomechanical changes and clarify the causal relationships underlying these adaptations. Third, examining older populations, who often experience FHP [33, 34] and have higher fall risks [9], would help determine whether the biomechanical adaptations observed in younger, healthy adults extend to more vulnerable groups. This line of inquiry could guide targeted assessments and interventions tailored to the needs of older individuals.

The findings of this study offer the following practical applications. First, the observed gait adaptations in participants with FHP emphasize the interconnectedness of head, neck, and other body segments. This finding underscores the need to consider whole-body mechanics when addressing FHP. Second, functional assessments for individuals with FHP should incorporate evaluations of trunk segment adaptations during walking. The compensatory trunk flexion observed during specific gait phases highlights the importance of trunk biomechanics in maintaining gait stability and efficiency. These insights can inform the development of comprehensive assessments and interventions targeting both postural and gait-related changes. Lastly, the study proposed a performance-based CVA cut-off as an objective criterion for screening individuals with FHP. This cut-off provides a practical alternative in contexts where standardized criteria are unavailable [12].

## Conclusion

This study found that, while spatiotemporal gait parameters and COM trajectories were similar between FHP and control groups, participants with FHP exhibited increased trunk flexion during specific phases of the gait cycle. A CVA cut-off of 44 degrees is proposed as a potential criterion for FHP diagnosis. These findings suggest compensatory trunk flexion strategies in individuals with FHP and offer insights into the biomechanical adaptations associated with FHP.

## Electronic supplementary material

Below is the link to the electronic supplementary material.

Supplementary Material 1

Supplementary Material 2

## Data Availability

The datasets used and analyzed during the current study are available from the corresponding authors upon reasonable request.
