# Supplementary figures and images for: Gait biomechanics and postural adaptations in forward head posture: a comparative cross-sectional study
Source: BMC Musculoskelet Disord. 2025 Aug 7;26:754. doi: 10.1186/s12891-025-08882-8 (PMC12329986; doi:10.1186/s12891-025-08882-8)

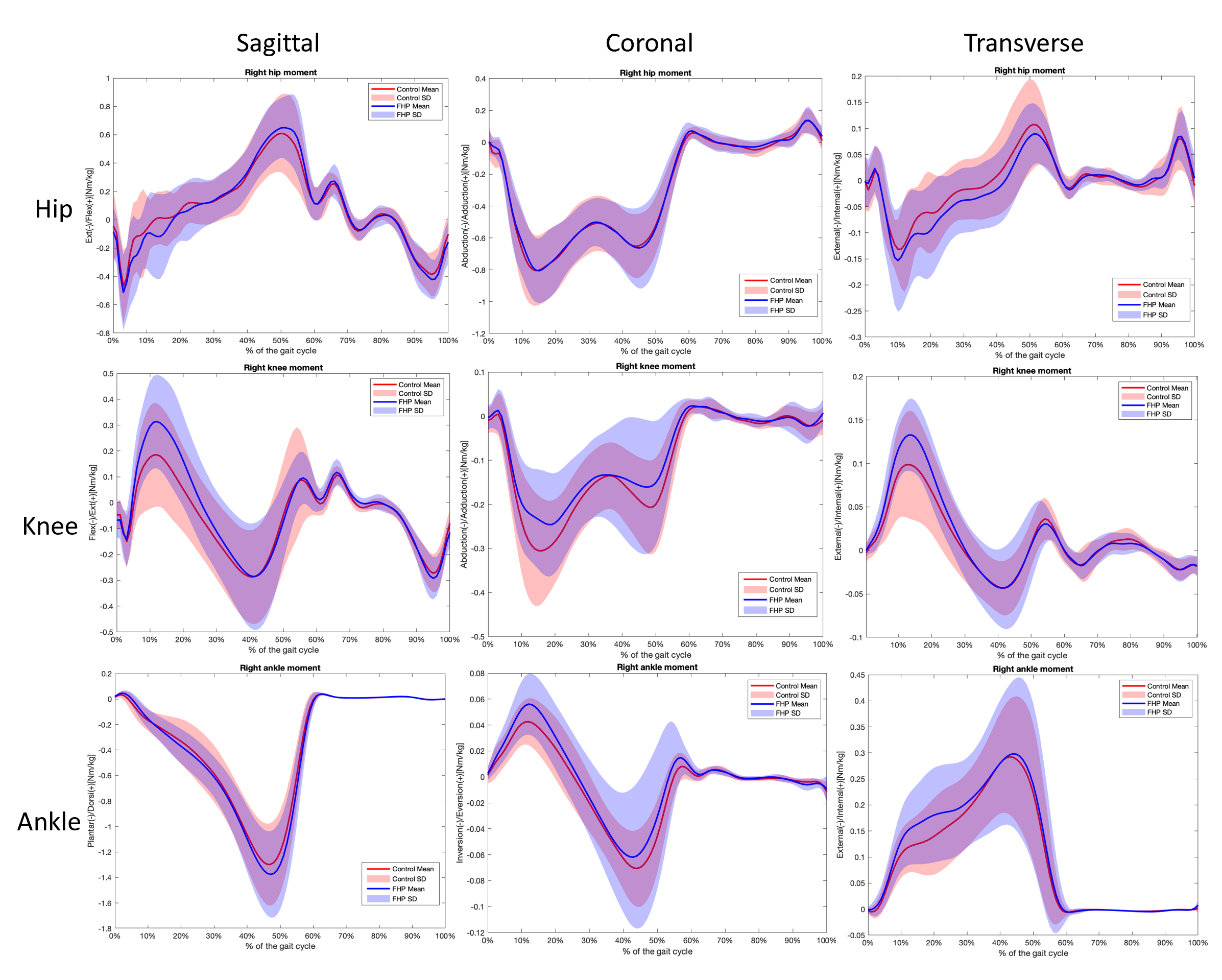

Supplement: Supplementary file 1 — Supplementary Material 1 [file 12891_2025_8882_MOESM1_ESM.tif]
