# Supplementary material for: Gait biomechanics and postural adaptations in forward head posture: a comparative cross-sectional study
Source: BMC Musculoskelet Disord. 2025 Aug 7;26:754. doi: 10.1186/s12891-025-08882-8 (PMC12329986; doi:10.1186/s12891-025-08882-8)
Supplement: Supplementary file 2 — Supplementary Material 2 [file 12891_2025_8882_MOESM2_ESM.docx]

**
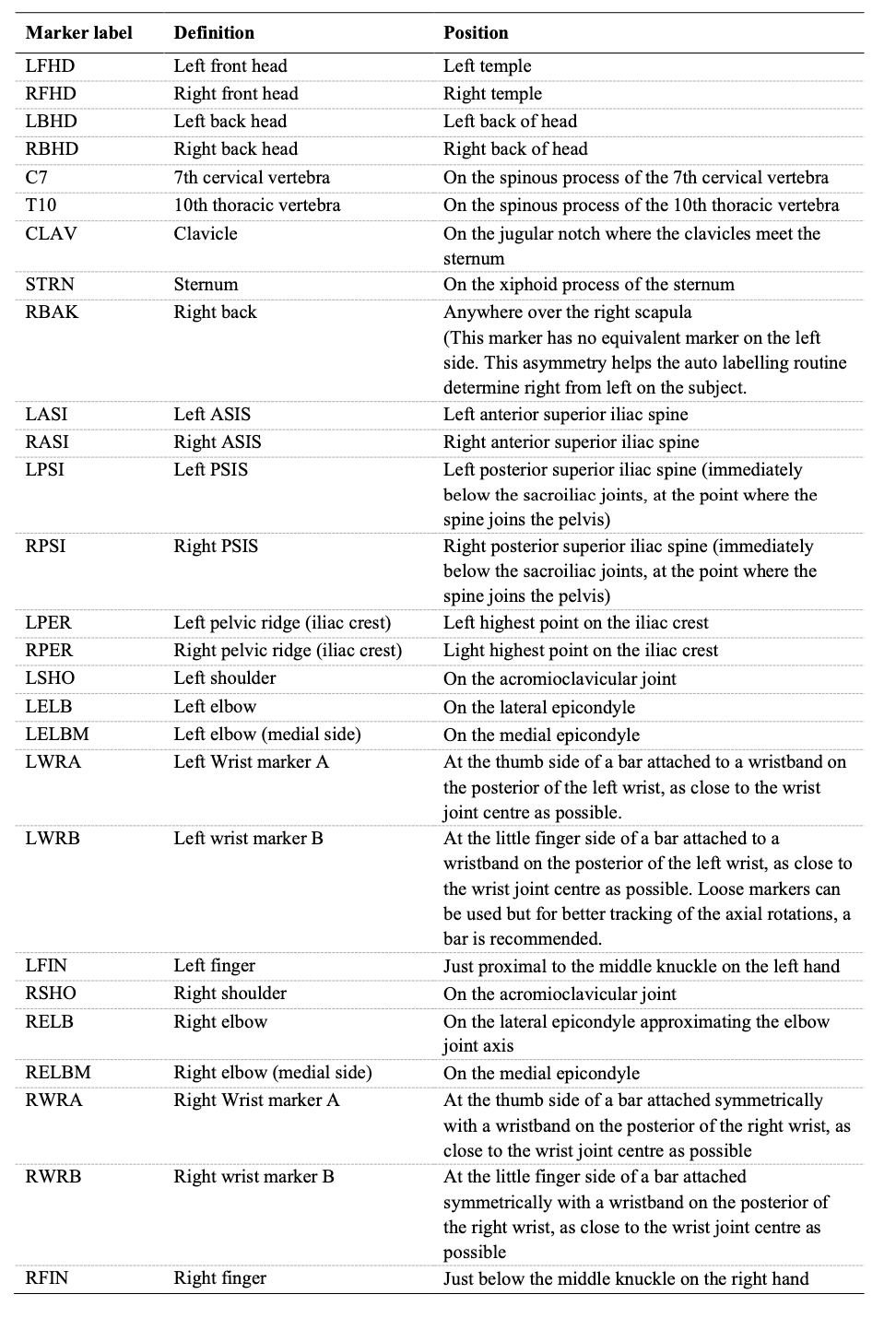
****Supplementary Table** The details of the full-body marker set.

**
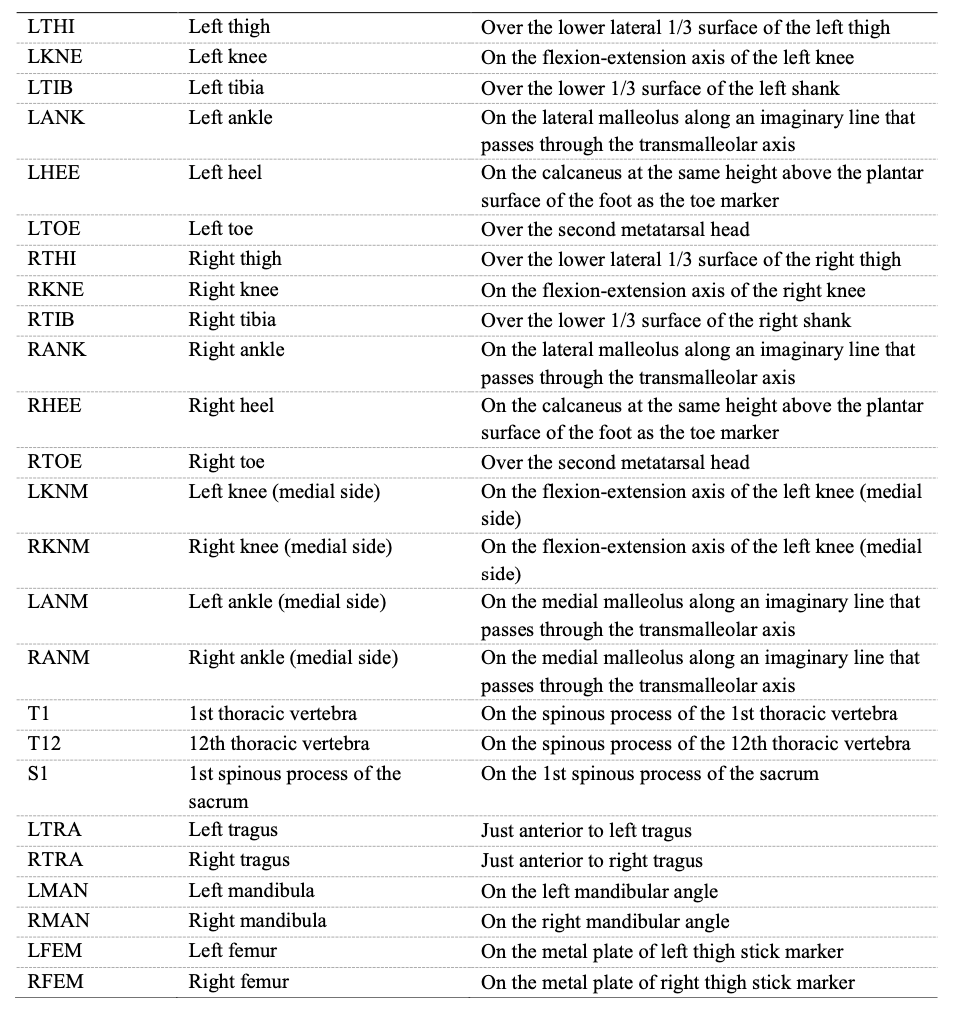
***The full-body marker set referred to the VICON Plug-in Gait reference guide, incorporating the additional markers.
